# Effect of pore size and shape on the thermal conductivity of metal-organic frameworks†
†Electronic supplementary information (ESI) available. See DOI: 10.1039/c6sc03704f



**DOI:** 10.1039/c6sc03704f

**Published:** 2016-09-07

**Authors:** Hasan Babaei, Alan J. H. McGaughey, Christopher E. Wilmer

**Affiliations:** a Department of Chemical & Petroleum Engineering , University of Pittsburgh , 3700 O'Hara St , Pittsburgh , PA 15261 , USA . Email: hasan.babaei@pitt.edu; b Department of Mechanical Engineering , Carnegie Mellon University , 5000 Forbes Avenue , Pittsburgh , PA 15213 , USA

## Abstract

We investigate the effect of pore size and shape on the thermal conductivity of a series of idealized metal-organic frameworks (MOFs) containing adsorbed gas using molecular simulations. With no gas present, the thermal conductivity decreases with increasing pore size. In the presence of adsorbed gas, MOFs with smaller pores experience reduced thermal conductivity due to phonon scattering introduced by gas–crystal interactions. In contrast, for larger pores (>1.7 nm), the adsorbed gas does not significantly affect thermal conductivity. This difference is due to the decreased probability of gas–crystal collisions in larger pore structures. In contrast to MOFs with simple cubic pores, the thermal conductivity in structures with triangular and hexagonal pore channels exhibits significant anisotropy. For different pore geometries at the same atomic density, hexagonal channel MOFs have both the highest and lowest thermal conductivities, along and across the channel direction, respectively. In the triangular and hexagonal channeled structures, the presence of gas molecules has different effects on thermal conductivity along different crystallographic directions.

## Abstract 2

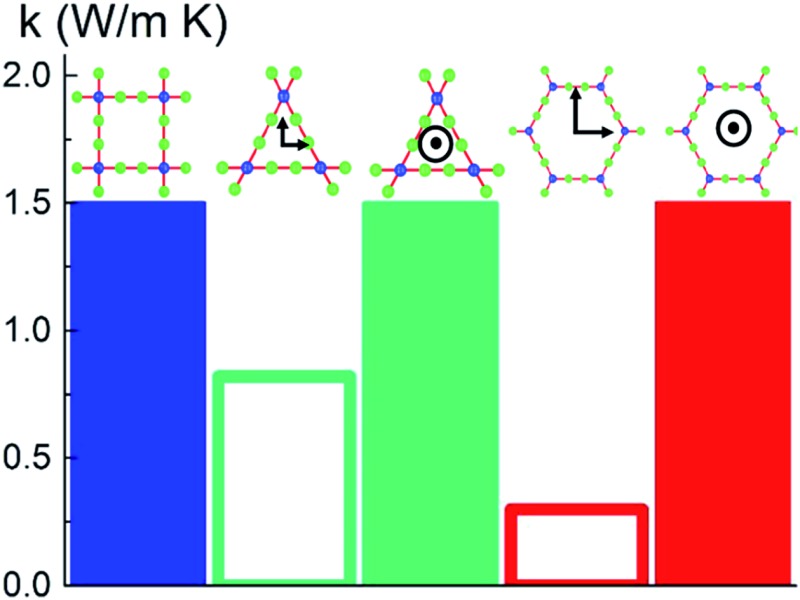
We investigate the effect of pore size and shape on the thermal conductivity of a series of idealized metal-organic frameworks (MOFs) containing adsorbed gas using molecular simulations.

## Introduction

Metal-organic frameworks (MOFs) have exceptional adsorption properties and show promise for applications such as gas storage, gas separation, and catalysis.^1–4^ Challenges remain, however, related to the rate at which gases can be loaded into MOFs without causing sharp temperature rises due to the heat generated during adsorption. This issue is an important consideration in applications such as natural gas storage in passenger vehicles, where tanks need to be refilled quickly to compete with traditional gasoline/diesel-based vehicles. Similarly, sharp drops in temperature during unloading can be a concern for sorption-based heat pumps,^5,6^ where the working fluid needs to be both adsorbed and desorbed quickly. To dissipate/recover the generated/lost heat quickly and thereby mitigate sharp temperature rises/drops requires the adsorbent to have a high thermal conductivity. Despite the importance of heat transfer in MOFs, however, studies of their thermal transport properties are limited,^7–12^ with only two that considered heat transfer in the presence of an adsorbed gas.^13,14^

The large number of MOFs already synthetized^1^ and the practically infinite number of MOFs not yet synthetized^15^ promise a large design space for thermal properties (*e.g.*, thermal conductivity, heat capacity, thermal expansion coefficient, and thermal stability). The key to efficiently exploring such a design space is an understanding of the relevant structure–property relationships. In this regard, molecular modeling can help by predicting the thermal properties of existing and future MOFs. Screening potential MOFs to find those with the right combination of thermal properties, pore size and shape, type and size of organic ligands, and internal surface area will help others decide which structure best suits their application need.^15^

In this paper, we address the effect of pore size and shape on the thermal conductivity of MOFs. We perform molecular dynamics (MD) simulations on a series of idealized model structures and apply the Green–Kubo method to predict their thermal conductivities with and without adsorbed gas at a range of densities. We find that MOF thermal conductivity decreases with increasing pore size and that the presence of adsorbed gas has a more severe effect in reducing the thermal conductivity of MOFs with smaller pores. We predict anisotropic thermal conductivity in MOFs with triangular and hexagonal channels and find that adsorbed gas affects thermal transport along the orthogonal directions differently, indicating a strong dependency of thermal transport on the mobility of gas molecules in those directions.

## Methodology

**Fig. 1:**
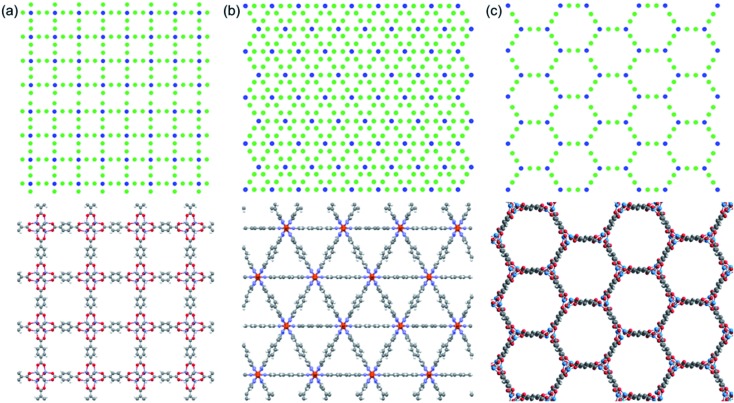
Cross-sectional views of (a) the idealized simple cubic structure (top) based on the real material IRMOF-1 (bottom), (b) the idealized triangular-channel structure (top) based on the real material FeBDP (bottom), and (c) the idealized hexagonal-channel structure (top) based on the real material MOF-74 (bottom).

We use idealized MOF structures with different pore shapes, as shown in Fig. 1a (cubic pores), 1b (triangular channels), and 1c (hexagonal channels). These structures are inspired by IRMOF-1,^16^ FeBDP,^17^ and MOF-74.^18^ The simple cubic lattice structures are built using different number of atoms per unit cell (7, 10, 13, 16, 19, and 22) to create different pore sizes (1.0, 1.3, 1.7, 2.0, 2.3, and 2.7 nm). The structures with triangular and hexagonal channels are built from parallel planes that contain a triangular or hexagonal lattice. For both of these structures, the spacing between the planes and between the lattice points on each plane is 1 nm. The atoms sitting at the lattice points are connected through two linker atoms in the directions both parallel and normal to the plane.

**Fig. 2:**
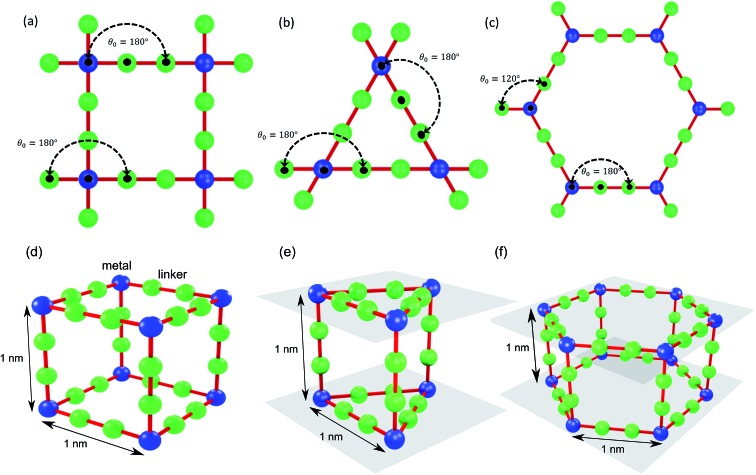
Bonds and angles for the unit cells of (a) simple cubic, (b) triangular-channel, and (c) hexagonal-channel structures. (d)–(f) Spacing between lattice points and planes for the cubic, triangular-channel and hexagonal-channel structures.

As depicted in Fig. 2, we define two-body bonded and three-body angular interactions between atoms, which are modeled using the class 2 quartic^19^ (bonds) and harmonic (angles) potentials. The force field parameters (see ESI†) were chosen so that the thermal conductivity of the simple cubic structure with a pore size of 1 nm was of the same order as typical MOFs (∼1 W m^–1^ K^–1^). For all angle bending potentials, we used an equilibrium angle *θ*_0_ of 180° except for the angles containing the corner atoms of the hexagonal structure, for which an equilibrium angle *θ*_0_ of 120° was used.

The gas is methane, which is modeled as a point particle. The initial configurations for the MD simulations of gas-loaded MOFs were taken from snapshots of equilibrated grand canonical Monte Carlo (GCMC) simulations^20^ (see ESI†), giving rise to gas densities from 0 to 12 molecules per nm^3^. Framework atoms were held fixed at their crystallographic coordinates in the GCMC calculations.

We applied the Green–Kubo method to predict thermal conductivity,^21^ which is based on calculating the instantaneous heat flux in an equilibrium MD simulation. All simulations were carried out at a temperature of 300 K and atmospheric pressure using a time step of 1 fs. The partial enthalpy terms required to analyze multicomponent systems were implemented as discussed in ref. 21–23. To gain further insight into the thermal conductivity predictions, we also calculated the corrected diffusivity of gas molecules within the MOFs, which is associated with the molecular mobility.^20^ The corrected diffusivity is based on a Green–Kubo relation and is defined as the time integral of the center of mass velocity autocorrelation function for the gas component. Details of the Green–Kubo calculations for both thermal conductivity and diffusivity along with samples of the associated autocorrelation functions and their integrals are provided in the ESI.†

## Results and discussion

### Effect of pore size

**Fig. 3:**
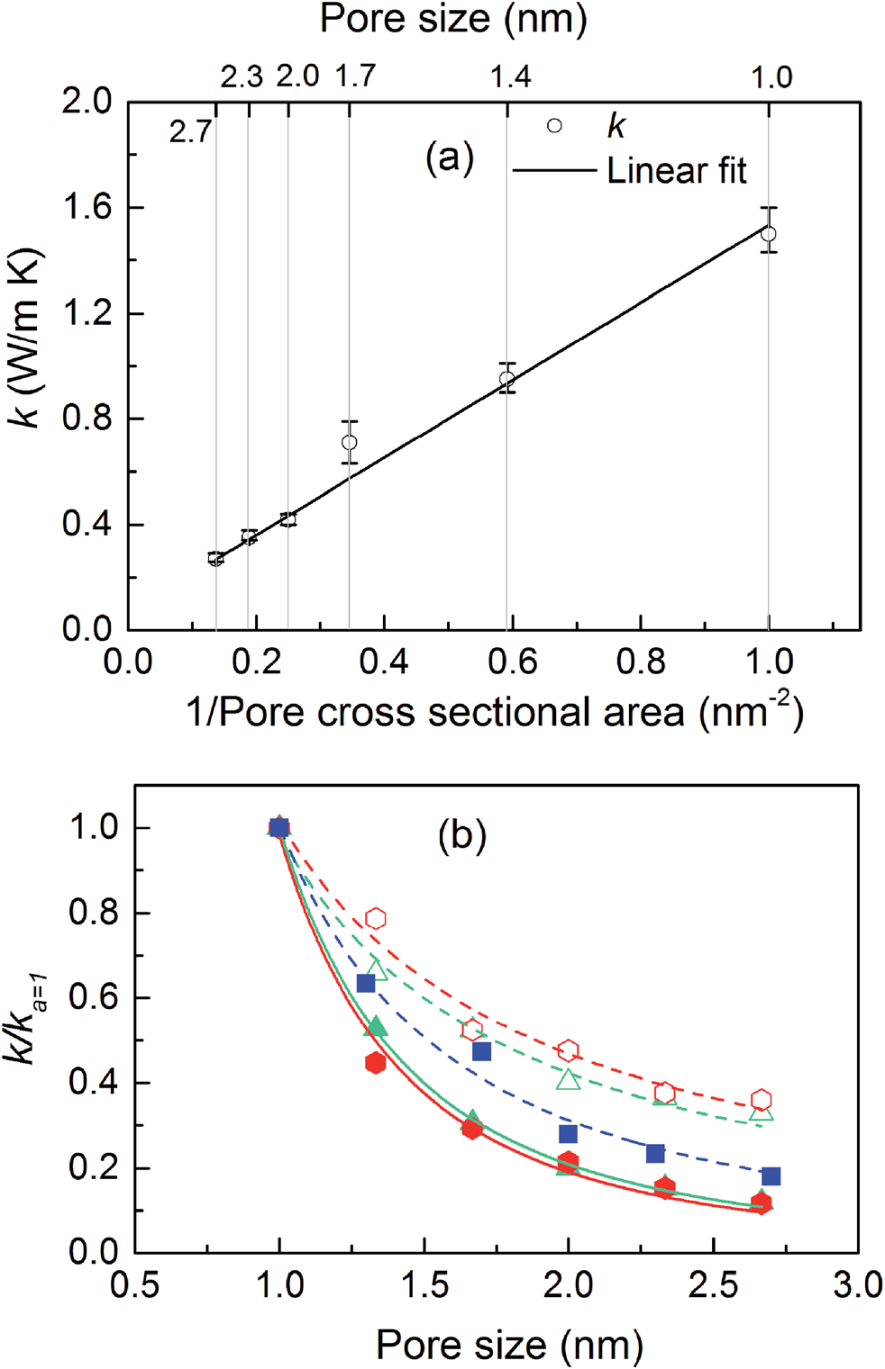
(a) Thermal conductivity of the simple cubic structure without adsorbed gas as a function of pore size (top axis) and crystal density (bottom axis). (b) Thermal conductivity of MOF crystals triangular (triangles) and hexagonal (hexagons) channels parallel (filled symbols) and perpendicular (empty symbols) to the channel directions along with simple cubic structure (filled squares) as a function of pore size. The lines in (b) are power law fits to the data points with exponents: squares: –1.78, filled hexagons: –2.13, filled triangles: –2.19, empty hexagons: –1.07 and empty triangles: –1.14.

The thermal conductivities of the structures with cubic pores were first predicted without any adsorbed gas. As shown in Fig. 3a, thermal conductivity decreases as the pore size increases. This trend is likely due to the decreased areal density of bonded interactions. In the absence of gas, these bonds are the means of transporting heat through the atomic vibrations (*i.e.*, phonons) in a dielectric solid. Supporting this argument, as also shown in Fig. 3a, thermal conductivity increases as the inverse of pore cross sectional area (which is proportional to the number of bonded interactions crossing per unit area) increases. Interestingly, this increase in thermal conductivity is a linear function of the inverse of the pore cross sectional area.

The thermal conductivities of triangular- and hexagonal-channel MOFs along with the simple cubic structures, normalized to that of the corresponding MOFs with 1 nm pores, are plotted *versus* pore size in Fig. 3b (absolute values are given in the ESI†). The interplanar distance was fixed at 1 nm. Thermal conductivity perpendicular to the channel direction, for both triangular and hexagonal channels, decreases as the inverse of the pore size, while thermal conductivity parallel to the channel direction decreases as the inverse of pore size squared. This finding supports the dependency of thermal conductivity on the areal density of bonds parallel to that direction.

**Fig. 4:**
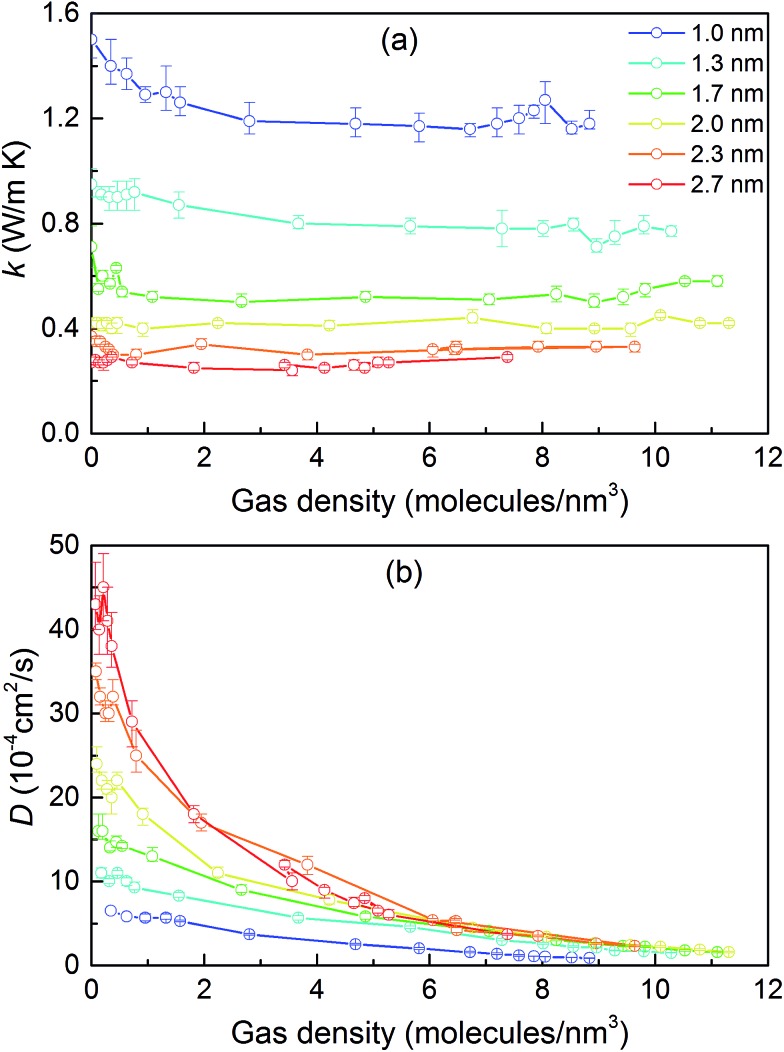
(a) Thermal conductivity of gas-loaded simple cubic MOFs across a range of pore sizes as a function of gas density. (b) Corrected diffusivity of gas molecules for the same structures as a function of gas density. In both (a) and (b), the connecting lines are used so that the data points from each series are better distinguished; they are not intended to indicate any trend.

We then predicted the thermal conductivity of the cubic pore materials loaded with gas at densities between 0 and 12 molecules per nm^3^. The results are plotted in Fig. 4a. At the maximum gas pressure considered, each structure (depending on the pore size) yielded a different gas density, which is why not all of the curves have the same maximum density. For structures with large pores (2.3 and 2.7 nm), increasing the gas density beyond the maximum density given in the curves led to a collapse. For pores up to 1.7 nm in size, increasing the gas density from 0 to 6 molecules per nm^3^ causes the thermal conductivity to decrease. Increasing the gas density further results in an increase in thermal conductivity, a trend similar to that observed in ref. 13, followed by a small drop. We verified that this drop is not due to statistical uncertainty by repeating our calculations and averaging over more simulations than the other cases (twelve instead of eight) that each had four times as many time steps. Through phonon lifetime calculations, we showed in ref. 13 that the initial decrease is due to phonon scattering processes introduced by collisions between gas molecules and the lattice. The increase in thermal conductivity at larger gas densities is related to the increased thermal conductivity of the gas itself. The final drop and rise in thermal conductivity (observed clearly for the structures with 1.0 nm and 1.3 nm pores at ∼8 and ∼8.5 molecules per nm^3^ and slightly for the structure with 1.7 nm pores at ∼8.5 molecules per nm^3^) is a phenomenon for which we do not have a clear understanding. For structures with pores larger than 1.7 nm, the thermal conductivity is nearly constant over all gas densities, all falling within 0.03 W m^–1^ K^–1^ of their average value. The uncertainty in the predicted thermal conductivities is 12%. This result implies that gas–crystal interactions for these structures are not effective in reducing thermal conductivity.

To better understand the gas molecule dynamics inside the pores, we calculated corrected gas diffusivities, which are plotted in Fig. 4b. For all pore sizes, the diffusivity decreases as the gas loading increases. The diffusivities for larger pores are generally higher compared to smaller pores. This finding indicates that for larger pores, at any given time, only a small fraction of gas molecules are interacting with the solid. One general challenge in these systems is how to define a collision for a molecule that spends most of its time interacting with the pore walls such that no sudden change is observable in its trajectory and associated quantities (*e.g.*, velocity, potential energy, or force). To quantify this behavior, we predicted a collision time *τ* (*i.e.*, the average time between collisions with the solid for a molecule) by performing MD simulations of a single gas molecule inside the MOF and using a Green–Kubo-based formula defined as1
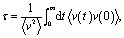
where *v*(*t*) is the molecule's velocity at time *t* and *v* ^2^ is the time-average of its squared velocity. As described in the ESI,† this equation is derived by assuming a bulk gas system, but it is qualitatively applicable for gas molecules inside a MOF. A sample of the velocity autocorrelation function and its integral for a gas molecule moving inside a MOF is provided in the ESI.† Qualitatively, the autocorrelation function shows how the instantaneous velocity of the gas molecule is correlated to its past velocities. A higher correlation implies less frequent changes in the velocity of gas molecule, which, in the single gas-MOF system, can only originate from collisions with crystal sites. A longer collision time indicates that the molecule undergoes fewer collisions during its motion inside the MOF.

**Fig. 5:**
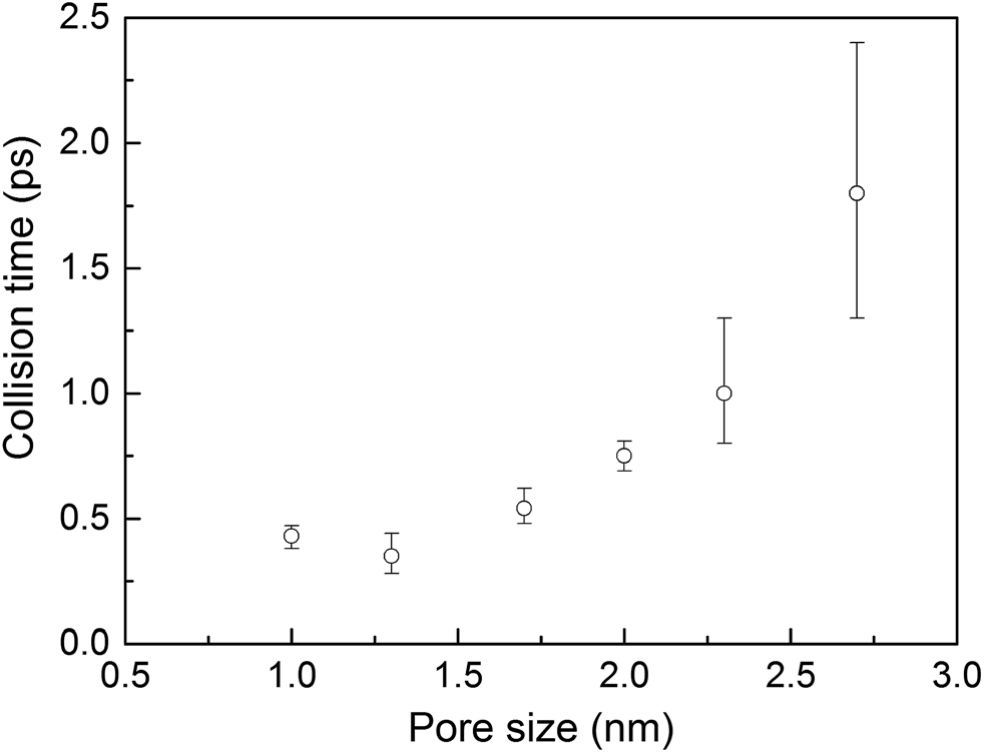
Average collision time for a gas molecule inside the simple cubic MOF *vs.* pore size.

Collision times obtained from eqn (1) are plotted as a function of pore size in Fig. 5. Supporting our hypothesis, the collision time increases as pore size increases. We note that the collision times for pore sizes of 1.0 and 1.3 nm are within their uncertainties. We also tracked the collisions for an isolated gas molecule by examining the time evolution of its potential energy, which increases when it approaches the pore. Plots for different pore sizes are provided in Fig. S7 of the ESI.† As shown there, there are higher fluctuations in the potential energy for smaller pores, also indicating that the collision time increases as the pore size increases. For bigger pores, the probability of collisions is smaller, such that this mechanism is not as effective at reducing thermal conductivity as it is for small pores. This finding is consistent with the insignificant change in the thermal conductivity of the MOFs with larger pores, as shown in Fig. 4a.

The calculated thermal conductivities for MOFs with different pore sizes indicate that small pores lead to better heat transfer and, therefore, are more efficient for rapid gas storage applications. While thermal conductivity reduction due to gas adsorption is more severe for MOFs with smaller pores, they are still better for gas storage. For example, the lowest thermal conductivity for a gas-loaded MOF with 1 nm pores is three times higher than the thermal conductivity for a MOF with 2.7 nm pores (Fig. 4a).

### Effect of pore shape

**Fig. 6:**
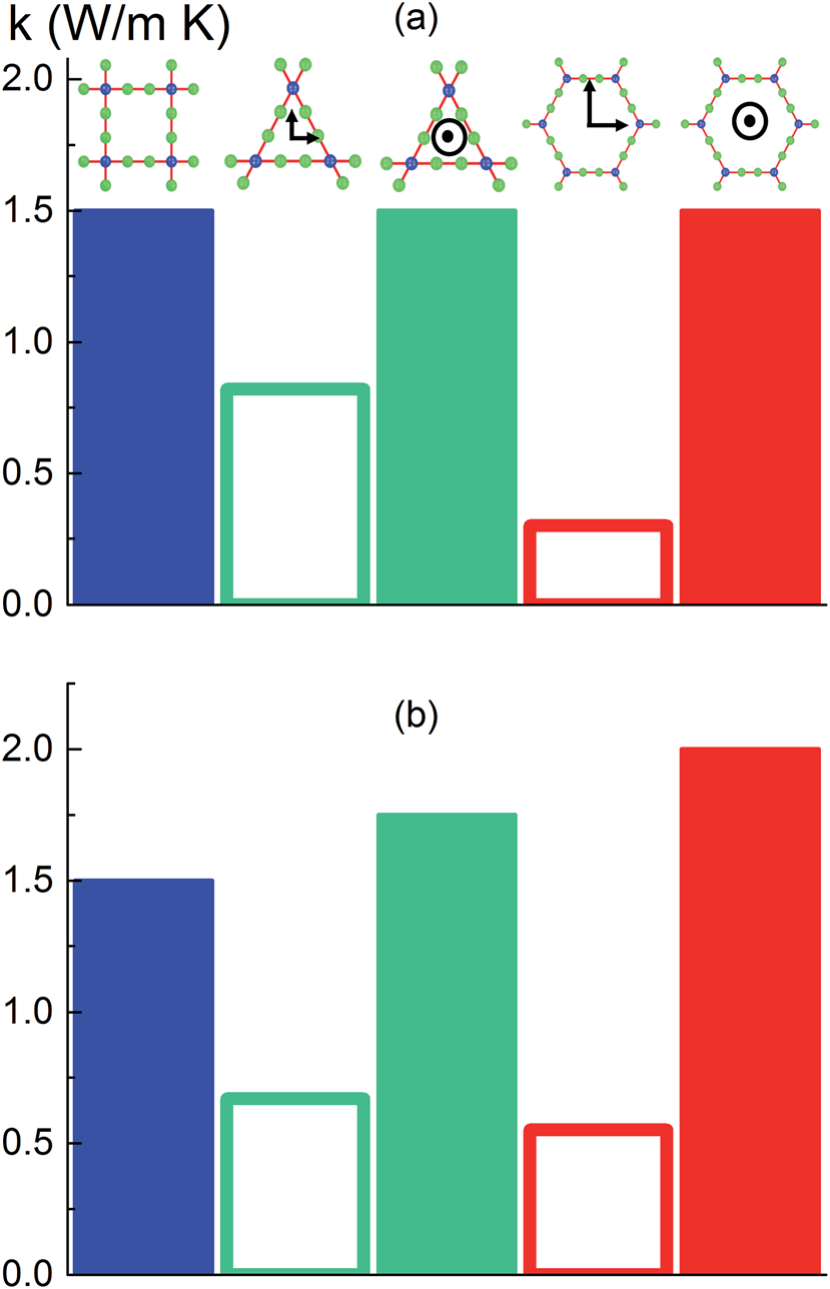
Calculated thermal conductivities of MOF structures with different pore shapes with the same (a) linker size (1 nm) and (b) cross sectional area (1 nm^2^). Blue bar: simple cubic structure. Green bars: triangular-channel structure (open bar: perpendicular to the channel, filled bar: parallel to the channel). Red bars: hexagonal-structure (open bar: perpendicular to the channel, filled bar: parallel to the channel).

To investigate the effect of pore shape on thermal conductivity, we next consider the MOFs with triangular and hexagonal channels, all with linker size of 1 nm. Due to their structural anisotropy, thermal conductivities perpendicular and parallel to the channels were calculated. As shown in Fig. 6a, the thermal conductivity in the parallel direction is the same for both structures (and the same as the isotropic value for the simple cubic structure), whereas it is lower in the perpendicular direction. For the hexagonal-channel structure, the thermal conductivity anisotropy ratio is five, while for the triangular-channel structure it is two. In comparing thermal conductivities of different structures at the same atomic density, we note that the highest overall thermal conductivity is along the channel direction of the hexagonal-channel structure, while the lowest is normal to the channel direction of the hexagonal channels (values of thermal conductivity as a function of atomic density for MOFs with different pore shapes are given in Fig. S11 in the ESI†).

**Fig. 7:**
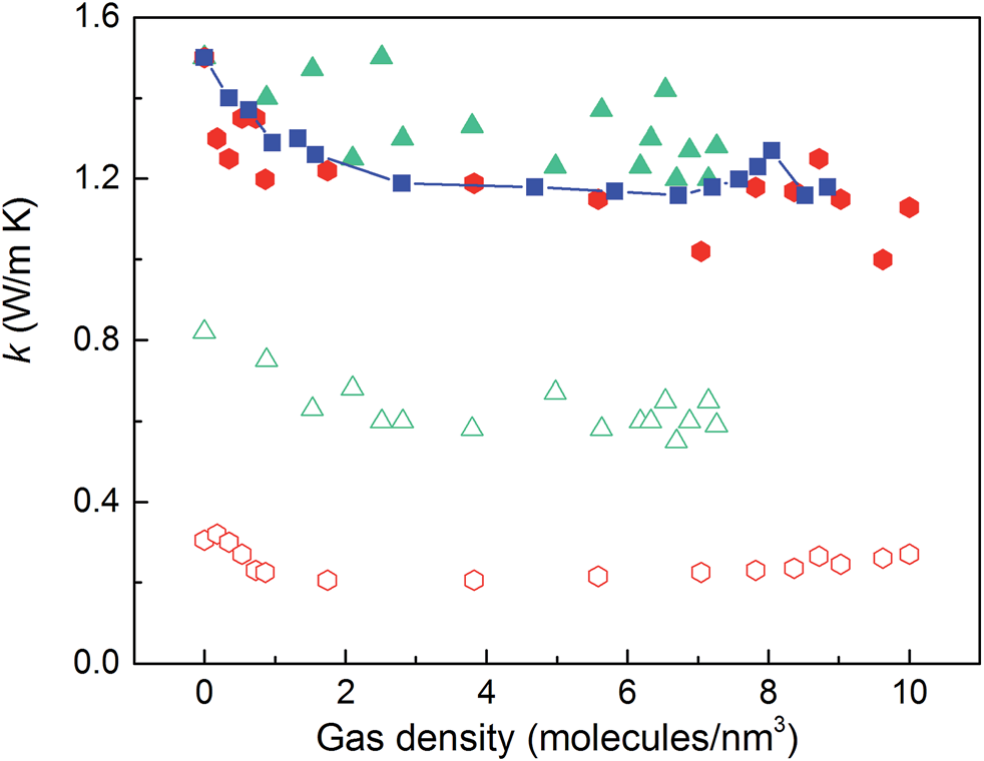
Thermal conductivity of gas-loaded MOF crystals with triangular (triangles) and hexagonal channels (hexagons) parallel (filled symbols) and perpendicular (empty symbols) to the channel directions. Squares: thermal conductivity of gas-loaded cubic structures.

The thermal conductivities of the structures with triangular and hexagonal channels with linker size of 1 nm in the presence of adsorbed gas are plotted in Fig. 7, along with the data for the simple cubic structure with a pore size of 1 nm from Fig. 4a. Similar to the simple cubic structure, the thermal conductivity of these two structures initially decreases with increasing gas density and then increases. In the perpendicular direction of the hexagonal structure, however, the rise in thermal conductivity starts at a smaller gas density (∼2 molecules per nm^3^) and rises more smoothly compared to the parallel direction and other structures (this trend is shown more clearly in Fig. S4 of the ESI†). The thermal conductivity increases from 0.21 to 0.27 W m^–1^ K^–1^ by increasing the gas density from 2 to 10 molecules per nm^3^. This behavior is due to the smaller value of thermal conductivity in the perpendicular direction and a relatively larger contribution of the gas component to the overall thermal conductivity. The increase in thermal conductivity at these gas densities is consistent with the increase in thermal conductivity for pure gas at the same gas densities (see Fig. S5 in ESI†). For example, by increasing gas density from 2 to 10 molecules per nm^3^, the rise in thermal conductivity for both pure gas and gas loaded MOFs is ∼0.06 W m^–1^ K^–1^.

The pores in the hexagonal-channel structure are larger than those in the triangular and cubic structures and so allow for more gas–gas interactions relative to gas–crystal interactions. We confirmed this behavior by calculating the diffusivities of gas molecules in different directions in each of the three structures (see Fig. S3 in ESI†). The diffusivities in the hexagonal-channel structure are the highest, showing a more bulk gas-like behavior with a higher probability of gas–gas interactions than gas–crystal interactions. It is also important to note that the diffusivity in the parallel to the channel direction of the triangular-channel structure is zero (*i.e.*, the pores are too small to allow gas flow in this direction). In this structure, gas molecules are trapped between the planes and can diffuse only in the perpendicular to the channel direction.

### Effect of pore shape at the same cross sectional area

Instead of keeping linker size the same for different pore shapes (previous section, see Fig. 6a), we now consider MOFs with the same pore cross sectional area. We designed triangular and hexagonal structures with a cross sectional area normal to the channel direction equal to that of the simple cubic structure (1 nm^2^). As shown in Fig. 6b, the parallel to the channel direction of hexagonal-channel MOF shows the highest thermal conductivity. This finding is consistent with the results for *k*/*ρ* (*ρ* is the MOF atomic density) for MOFs with different pore shapes (see the ESI†). The normal to channel direction thermal conductivity for the triangular structure is lower than the value for the same linker size case while for the hexagonal structure it is higher.

## Conclusion

With the purpose of understanding and ultimately improving heat transfer in MOFs, we investigated a series of idealized model systems using molecular simulations. We studied the effect of pore size and shape on thermal conductivity with and without adsorbed gases. As shown in Fig. 3a and b, we showed that the thermal conductivity of empty MOFs decreases with increasing pore size. For MOFs with pores smaller than ∼1.7 nm, the presence of gas molecules decreases their thermal conductivity due to phonon scattering introduced by gas–crystal interactions (see Fig. 4a). In contrast, for larger pores, the thermal conductivity does not change with increasing gas density. Using an adapted Green–Kubo-based approach, as shown in Fig. 5, we found a longer gas molecule–crystal collision for MOFs with larger pores, resulting in a lower frequency of gas–crystal collisions and consequently, less gas-induced phonon scattering.

Our study indicates that for applications in which rapid exchange of heat generated during adsorption is important (*e.g.*, gas storage), MOFs with smaller pores are likely to have better thermal performance. Our study also shows that MOFs with different pore shapes can exhibit significant anisotropy with regards to thermal transport (Fig. 6 and 7), suggesting that a strategy for rapidly adsorbing high concentrations of gas is to use structures with large channels (for capacity reasons) and short interplanar distances (for thermal conductivity reasons).
