# Supplementary material for: Effect of pore size and shape on the thermal conductivity of metal-organic frameworks
Source: Chem Sci. 2016 Sep 7;8(1):583–9. doi: 10.1039/c6sc03704f (PMC5358541; doi:10.1039/c6sc03704f)
Supplement: Supplementary file 1 [file SC-008-C6SC03704F-s001.pdf]

Electronic Supplementary Material (ESI) for Chemical Science.
This journal is © The Royal Society of Chemistry 2016

Supporting Information

Effect of pore size and shape on the thermal conductivity of metal-organic frameworks

Hasan Babaei,[a,b*] Christopher E. Wilmer[a] and Alan McGaughey[b]

[a]Department of Chemical & Petroleum Engineering, University of Pittsburgh, 3700 O'Hara St, Pittsburgh, PA, 15261

[b]Department of Mechanical Engineering, Carnegie Mellon University, 5000 Forbes Avenue, Pittsburgh, PA 15213, USA.

Contents

S1. Additional information on methodology, simulations, and sample data

S2. Collision time calculations and sample data

S3. Verification of independency of outcomes on metal-linker interactions

S4. Pore size effect on thermal conductivity of triangular and hexagonal structures

S5. k/density for different pore shapes

[*] Corresponding author. E-mail address: hasan.babaei@pitt.edu

S1. Additional information on methodology, simulations and sample data

Force field

As depicted in Figure 2 of the main paper, we define 2-body bonded and 3-body angular interactions between atoms, which are interpreted using the class2 quartic [$U(r) = K_2(r-r_0)^2 + K_3(r-r_0)^3 + K_4(r-r_0)^4$] [1] and harmonic [$U(\theta) = K(\theta-\theta_0)^2$] potentials. For bond stretching, we used a scaled set of parameters, borrowed from the parameters for sp3 carbon-carbon bonds[1] and divided by five (i.e., $K_2 = 60$ kcal/mol/Å^2, $K_3 = -100$ kcal/mol/Å^3, and $K_4 = 136$ kcal/mol/Å^4) and r_0 is the equilibrium distance between bonded atoms (3.34 Å). For all angle bending potentials, we used an equilibrium angle θ_0 of 180° and $K = 10$ kcal/mol/degree^2 except for the angles containing the corner atoms of the hexagonal structure for which an equilibrium angle θ_0 of 120° was used. These force field parameters were chosen so that the thermal conductivity of the simple cubic structure with pore size of 1 nm was of the same order as typical MOFs (~1 W/m K).

We chose a point particle with the TraPPE force field parameters[2] for methane as the model gas molecule. The Lennard-Jones (LJ) parameters for the atoms in the idealized structures (which were only used for interactions with gas molecules) were taken from the definition of carbon in the Universal Force Field (UFF).[3] In this study, a well depth of 2.57ε was used where ε is the well depth calculated in the standard way (i.e., the Lorentz–Berthelot mixing rules[4]). Based on our previous study, this choice of the energy scale results in adsorption isotherms similar to the materials of interest.

Green-Kubo calculation of thermal conductivity

The Green-Kubo relation for thermal conductivity is,[5]

$$k_{ii} = \frac{V}{k_B T^2}\int_0^{\infty} < J_i(t) J_i(0) > dt, \quad i = x, y \; or \; z. \tag{1}$$

In which the *i*-th diagonal element of the thermal conductivity tensor (k_{ii}) at temperature *T* is calculated by integrating over time the heat current autocorrelation function (HCACF). The HCACF is extracted from equilibrium molecular dynamics (MD) simulations. In Eq. (1), k_B is the Boltzmann constant, *V* is the volume of the simulation box that contains the system of particle,s and ***J***(t) is the microscopic heat current.

The microscopic heat current is calculated from[5]

$$\mathbf{J}(t) = \frac{1}{V}\left[\sum_{j=1}^{N}\mathbf{v}_j E_j - \sum_{\alpha=1}^{2} h_\alpha \sum_{j=1}^{N_\alpha}\mathbf{v}_{\alpha j}\right] + \frac{1}{V}\left[\frac{1}{2}\sum_{i=1}^{N}\sum_{j=1, j\neq i}^{N}\mathbf{r}_{ij}\left(\mathbf{v}_j . \mathbf{F}_{ij}\right)\right], \tag{2}$$

where $\mathbf{v}_j$ and E_j are the velocity vector and instantaneous energy of particle *j*. The quantities $\mathbf{r}_{ij}$ and $\mathbf{F}_{ij}$ are the displacement vector and interacting force between particles *i* and *j*. The parameter *N* is the total number of particles and N_α is the number of particles for species α. h_α denotes the average partial enthalpy of species α and is given by

$$h_\alpha = \frac{\sum_{i=1}^{N_\alpha}\left[K_i + U_i + \frac{1}{3}\left(m_i v_i^2 + \frac{1}{2}\sum_{j=1}^{N}\mathbf{r}_{ij} . \mathbf{F}_{ij}\right)\right]}{N_\alpha} \tag{3}$$

In which K_i and U_i are the time-averaged kinetic and potential energies of particles of species α.

For determining thermal conductivity, the time step for all simulations was 1 fs. The systems were initially equilibrated under *NVT* conditions for 300,000 time steps and further

equilibrated for 300,000 time steps under *NVE* conditions. Finally, the *NVE* simulations were run for an additional 1,000,000 time steps where the heat current was calculated every 5 fs. For all cases, we performed all procedures for eight simulations starting with random velocity distribution, which were then averaged for the thermal conductivity predictions. Thermal conductivity values were obtained from the plateau region of the HCACF integral [eq. (1)].

The initial atomic configurations for the MD simulations involving gases were taken from snapshots of equilibrated Grand canonical Monte Carlo (GCMC) calculations[6] at different pressures (described below). The framework atoms were fixed coordinates in the GCMC calculations.

A sample heat current autocorrelation and its integral are shown in Figure S1.

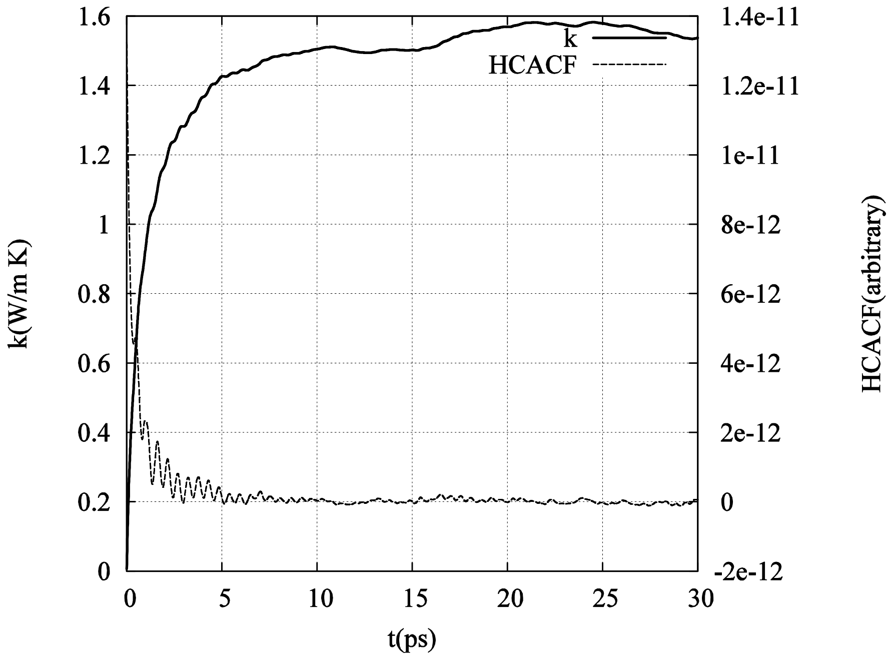

Figure S1 Samples of Green-Kubo calculations of thermal conductivity. Heat current autocorrelation and its time integral for the pure simple cubic model structure with pore size of 1 nm.

Green-Kubo calculation of corrected diffusivity

The Green-Kubo relation for diffusivity is[7]:

$$D = \frac{1}{N}\int_0^\infty dt \sum_{i=1}^{N}\sum_{j=1}^{N} < v_i(t) v_j(0) >, \qquad (4)$$

where N is the number of particles.

A sample of the velocity autocorrelation function and its integral is shown in Figure S2.

The corrected diffusivities for the triangular and hexagonal structures are shown in Figure S3. The diffusivities for the simple cubic structures with different pore sizes are shown in Figure 4 in the main paper.

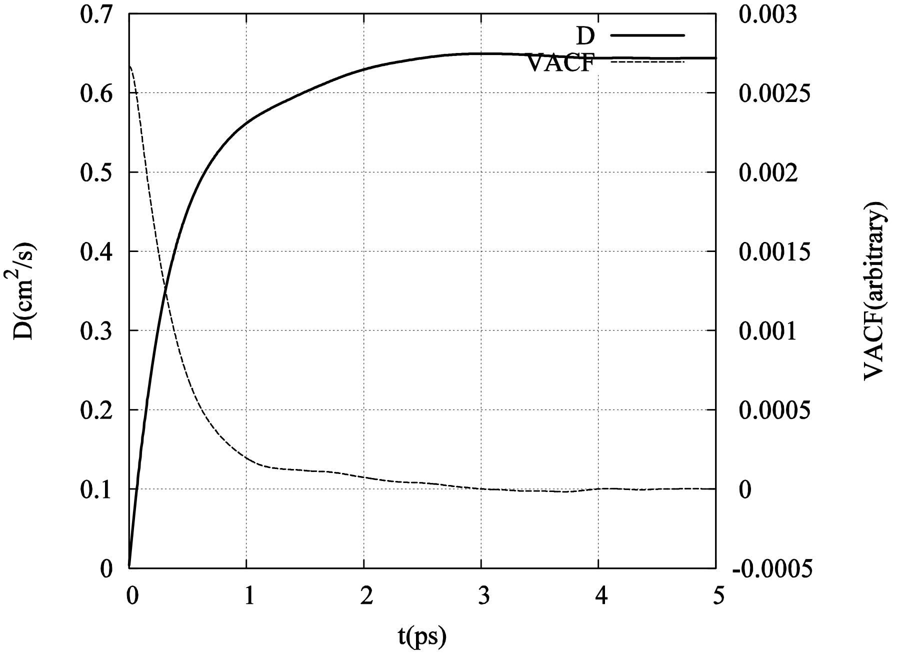

Figure S2 Sample of velocity autocorrelation function and its integral for the gas density of ~5 molecules/nm^3 in the cubic structure with pore size of 2.7 nm.

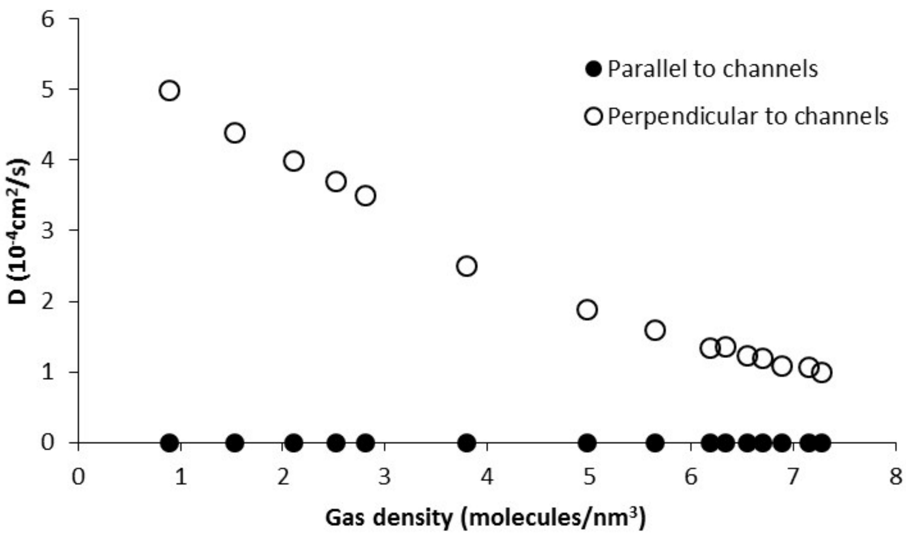

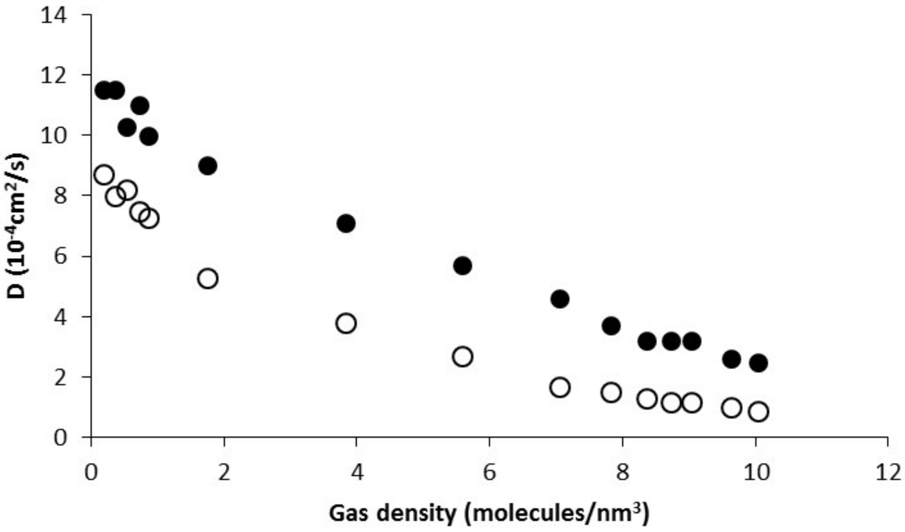

Figure S3 Gas diffusivity vs. density of loaded gas for the triangular (top) and hexagonal (bottom) structures along parallel and perpendicular to the channel directions.

Grand canonical Monte Carlo simulations

GCMC simulations were performed to estimate the adsorption of methane in the idealized MOFs. The interactions between non-bonded atoms were computed through the Lennard-Jones (LJ) potential:

$$V_{ij} = 4\varepsilon_{ij}\left[\left(\frac{\sigma_{ij}}{r_{ij}}\right)^{12} - \left(\frac{\sigma_{ij}}{r_{ij}}\right)^{6}\right] \qquad (5)$$

,

where i and j are interacting atoms, and r_{ij} is the distance between atoms i and j. ε_{ij} and σ_{ij} are the LJ well depth and diameter. The LJ parameters between atoms of different types were calculated using the Lorentz-Berthelot mixing rules (i.e., geometric average of well depths and arithmetic average of diameters). All GCMC simulations of methane adsorption included an M-cycle equilibration period followed by an M-cycle production run, where M was 5000. A cycle consists of n Monte Carlo steps; where n is equal to the number of molecules (which fluctuates during a GCMC simulation). All simulations included random insertion, deletion, and translation moves of molecules with equal probabilities. Atoms belonging to the crystal structure were held fixed at their crystallographic positions. An LJ cutoff distance of 12 Å was used for all simulations. Supercells containing 8x8x8 unit cells of crystals were used for the simulations. Methane adsorption was simulated at various pressures: 2, 4, 6, 8, 10, 20, 50, 100, 150, 200, 300, 400, 500, 600, 800, and 1000 bar, at 298 K. Fugacities needed to run the GCMC simulations were calculated using the Peng-Robinson equation of state.

Thermal conductivity of gas-loaded MOF crystal with hexagonal channel perpendicular to the channel direction

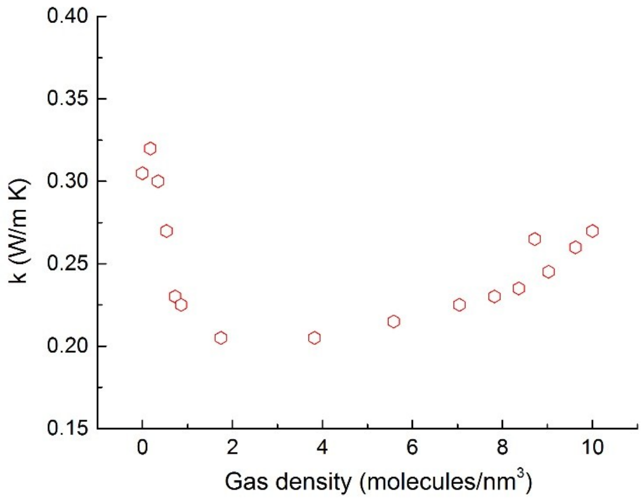

Figure S4 Thermal conductivity of gas-loaded MOF crystal with hexagonal channel perpendicular to the channel direction.

Thermal conductivity of pure gas vs. gas density

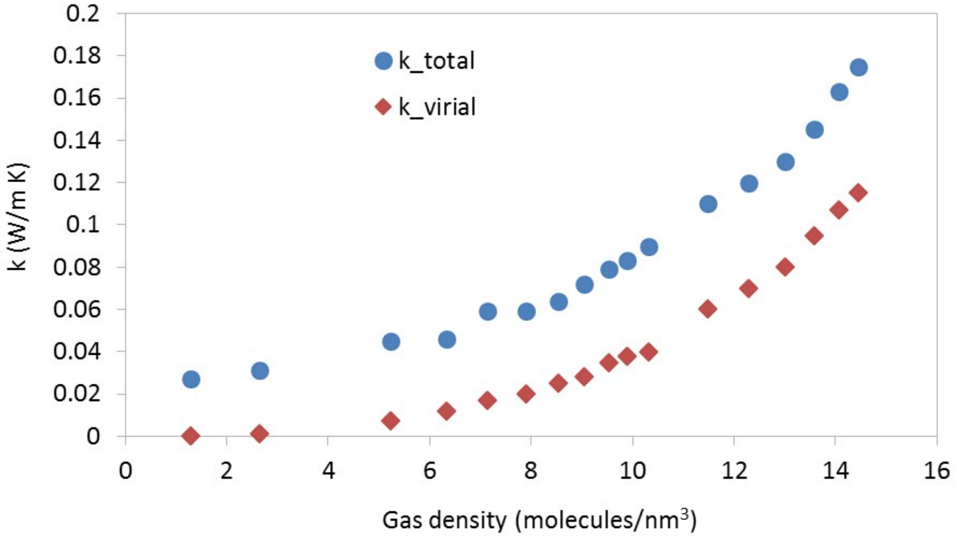

Figure S5 Thermal conductivity of pure gas vs. gas density.

S2. Collision time calculations and sample data

From fluctuation-dissipation theory (i.e., the Langevin equation), the self-diffusivity can be determined using the time integral of the autocorrelation of velocities as $\frac{1}{N}\int_0^\infty dt \sum_{i=1}^{N} < v_i(t) v_i(0) >$. On the other hand, from the kinetic theory, the self-diffusivity is equal to $< v^2 > \tau$, where τ is the average collision time for a gas molecule and $< v^2 >$ is the average of its squared velocity. Assuming an ideal gas (i.e., the correlation between velocities of different gas molecules can be ignored), we derive the following equation for the collision time:

$$\tau = \frac{1}{< v^2 >}\int_0^\infty dt < v(t) v(0) > \quad . \tag{6}$$

If we assume a similar stochastic dynamics for a single molecule inside the MOF, we can use the same equation to calculate collision times. A sample of the velocity autocorrelation function and its integral for a gas molecule moving inside a MOF is given in Figure S6. We should mention that the proposed method can only predict the exact value for collision time at low gas density where the gas-gas collisions are not important.

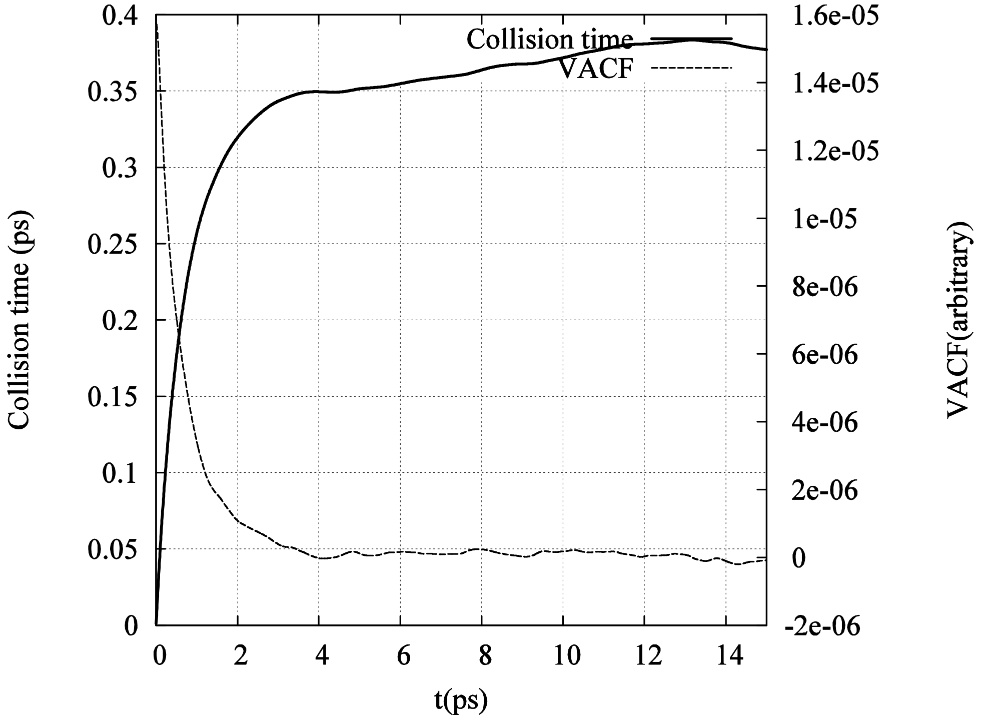

Figure S6 Sample of velocity autocorrelation function and its integral for a gas molecule moving inside cubic structure with pore size of 1 nm.

We can also record the potential energy (which changes with the distance to the pore walls) versus time to qualitatively shed light on the change of collision time with pore size. These potential energy plots for the simple cubic structures with different pore sizes are provided in Figure S7.

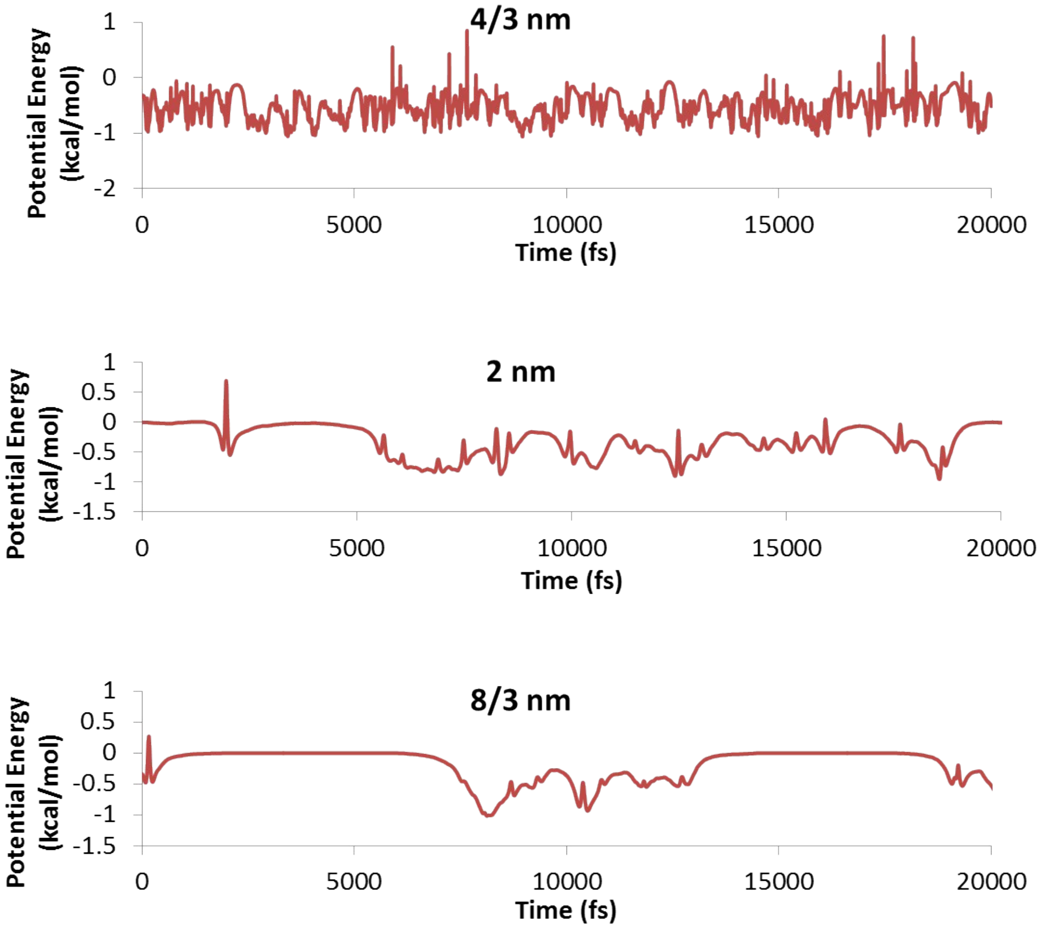

Figure S7 Potential energy vs. time for a single molecule moving in nanoporous simple cubic structures with different pore sizes.

S3. Verification of independency of outcomes on metal-linker interactions

To verify that the reported pore size and gas adsorption effects are independent of the interaction between metal atoms and linker atoms, we investigated two other models with weaker and stronger interaction between metal-linker than linker-linker atoms (spring constants equal to the 0.67 and 1.5 of the regular spring constants). The results (shown in the figures below) show consistency with the results presented in the main text. Thermal conductivity increases linearly with the inverse of pore cross sectional area (consistent with results presented in Figure 3a in the manuscript). Moreover, thermal conductivity decreases as the gas loading increases (consistent with results presented in Figure 4a in the manuscript).

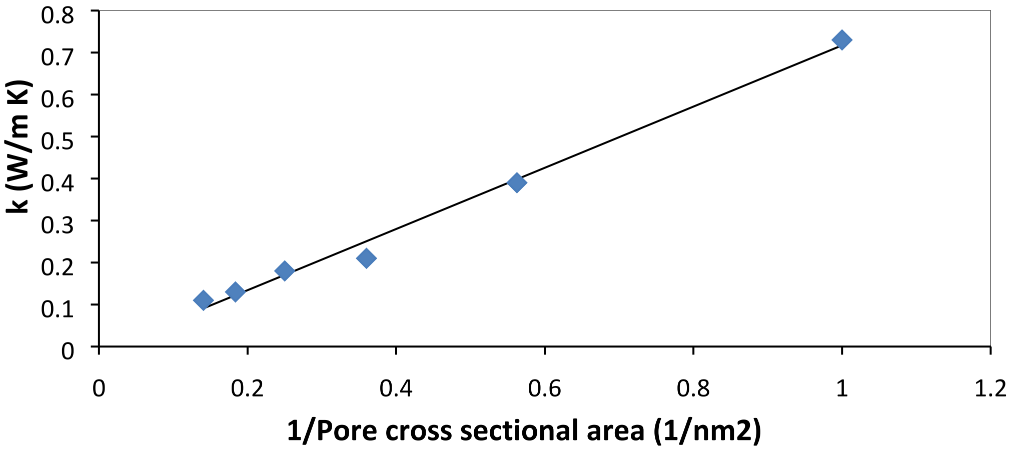

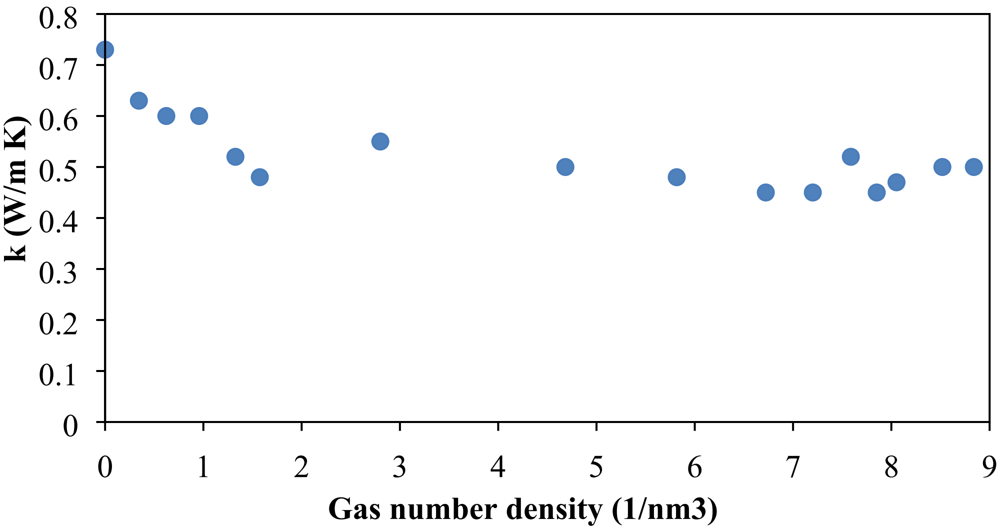

Figure S8 Pore size effect (top) thermal conductivity vs. gas density (bottom) for the 0.67 case

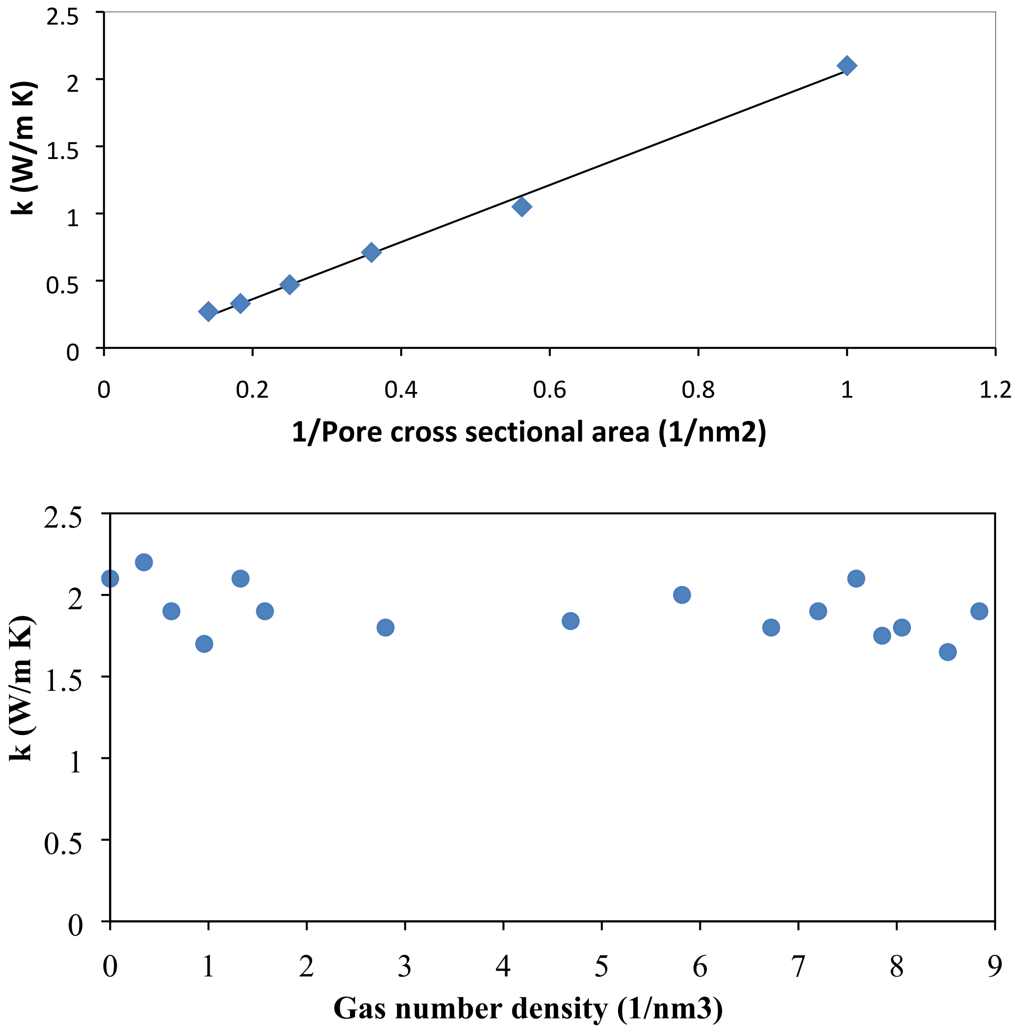

Figure S9 Pore size effect (top) thermal conductivity vs. gas density (bottom) for the 1.5 case

S4. Pore size effect on thermal conductivity of triangular and hexagonal structures

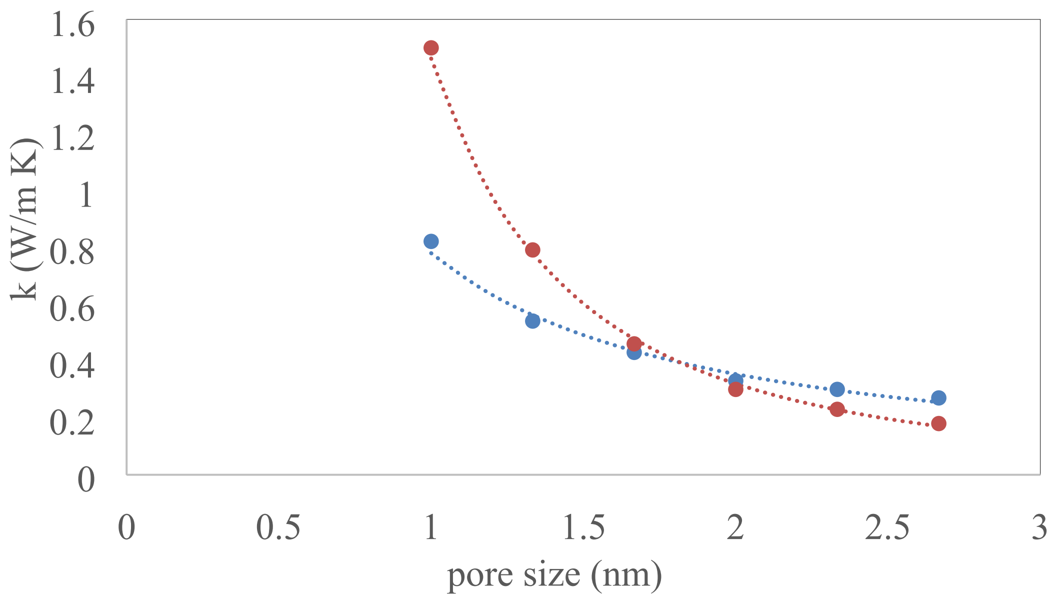

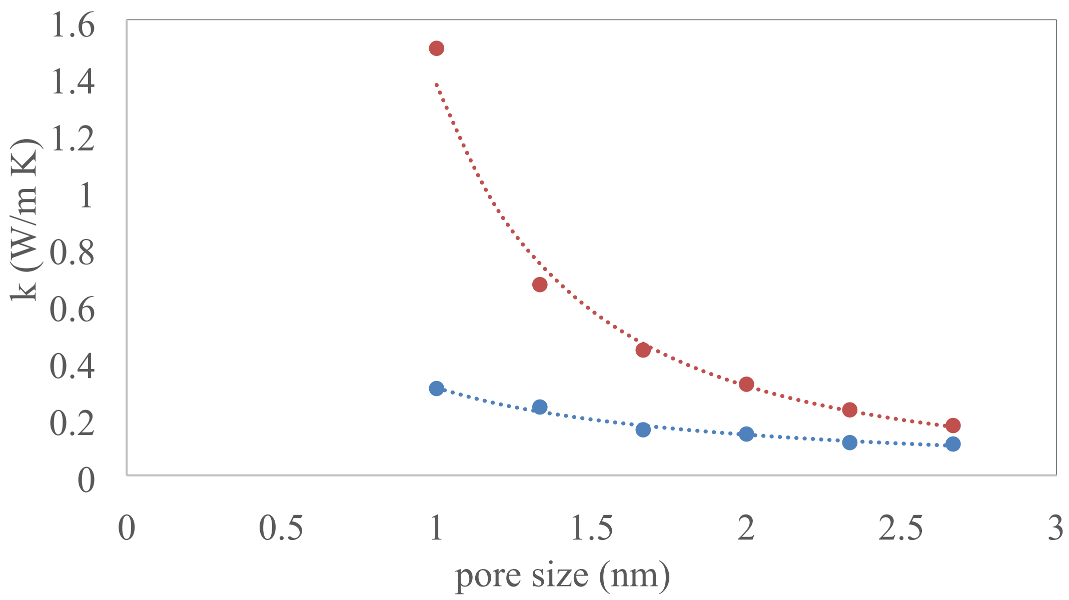

Figure S10 Pore size effect on triangular (top) and hexagonal (bottom) structures in parallel and normal to channel directions.

S5. k/density for different pore shapes

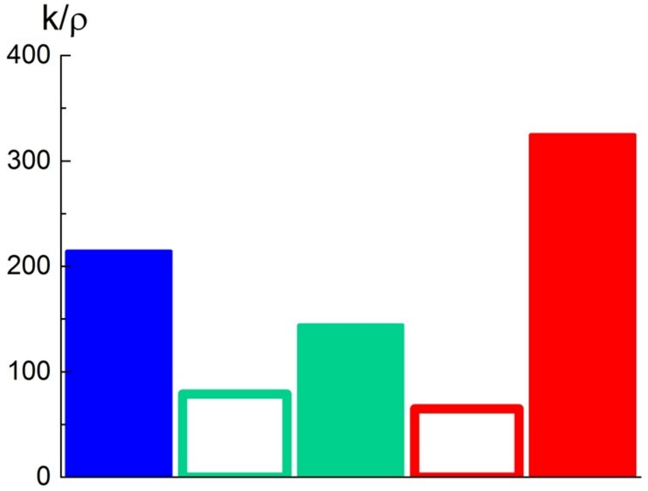

Figure S11 k/density for different pore shapes. Blue bar: cubic structure. Green bars: triangular-channel structure (open bar: perpendicular to the channel, filled bar: parallel to the channel). Red bars: hexagonal-structure (open bar: perpendicular to the channel, filled bar: parallel to the channel).

References

1 H. Sun, *J. Phys. Chem. B*, 1998, **102**, 7338–7364.
2 M. G. Martin and J. I. Siepmann, *J. Phys. Chem. B*, 1998, **102**, 2569–2577.
3 A. K. Rappe, C. J. Casewit, K. S. Colwell, W. A. Goddard and W. M. Skiff, *J. Am. Chem. Soc.*, 1992, **114**, 10024–10035.
4 M. P. Allen and D. J. Tildesley, *Computer Simulation of Liquids*, Oxford University Press, Oxford England; New York, Reprint edition., 1989.
5 H. Babaei, P. Keblinski and J. M. Khodadadi, *J. Appl. Phys.*, 2012, **112**, 054310.
6 R. Q. Snurr, A. T. Bell and D. N. Theodorou, *J. Phys. Chem.*, 1993, **97**, 13742–13752.
7 E. J. Maginn, A. T. Bell and D. N. Theodorou, *J. Phys. Chem.*, 1993, **97**, 4173–4181.
